# The common *FTO *variant rs9939609 is not associated with BMI in a longitudinal study on a cohort of Swedish men born 1920-1924

**DOI:** 10.1186/1471-2350-10-131

**Published:** 2009-12-09

**Authors:** Josefin A Jacobsson, Ulf Risérus, Tomas Axelsson, Lars Lannfelt, Helgi B Schiöth, Robert Fredriksson

**Affiliations:** 1 Department of Neuroscience, Functional Pharmacology, Uppsala University, Uppsala Sweden; 2 Department of Public Health and Caring Sciences, Uppsala University, Uppsala, Sweden; 3 Department of Medical Sciences, Uppsala University, Uppsala, Sweden

## Abstract

**Background:**

Common FTO (fat mass and obesity associated) gene variants have recently been strongly associated with body mass index and obesity in several large studies. Here we set out to examine the association of the *FTO *variant rs9939609 with BMI in a 32 year follow up study of men born 1920-1924. Moreover, we analyzed the effect of physical activity on the different genotypes.

**Methods:**

The *FTO *rs9936609 was genotyped using an Illumina golden gate assay. BMI was calculated using standard methods and body fat was estimated by measuring skinfold thickness using a Harpenden caliper. Physical activity was assessed using a four question medical questionnaire.

**Results:**

*FTO *rs9939609 was genotyped in 1153 elderly Swedish men taking part of a population-based cohort study, the ULSAM cohort. The risk of obesity and differences in BMI according to genotype at the ages of 50, 60, 70, 77 and 82 were investigated. We found no increased risk of obesity and no association with BMI at any age with the *FTO *rs9939609 variant. We found however interaction between physical activity at the age of 50 years and genotype on BMI levels (p = 0.039) and there was a clear trend towards larger BMI differences between the TT and AA carriers as well as between AT and AA carriers in the less physically active subjects.

**Conclusion:**

Here we found that the well established obesity risk allele for a common variant in *FTO *does not associate with increased BMI levels in a Swedish population of adult men which reached adulthood before the appearance of today's obesogenic enviroment. There is an interaction between physical activity and the effect of the FTO genotype on BMI levels suggesting that lack of physical activity is a requirement for an association of FTO gene variants to obesity.

## Background

Today, about one in three adult can be classified as obese based on objectively measured weight and height. The increase started after the World War II, escalated in the seventies and the obesity rates have roughly tripled in the past 20 years. In Sweden one in hundred is considered morbidly obese today compared to one in thousand in the early 1970s [1-4].

This worldwide rapid increase in the prevalence of obesity is much due to environmental factors, such as a sedentary lifestyle and develops from an imbalance between energy ingested and expended [5]. It is however also widely accepted that obesity is under strong genetic control, explaining about 30% to 70% of the variation in BMI [6-8]. Recently, genome-wide association studies have led to rapid progress in our understanding of the genetic basis of various common diseases and a new candidate gene for obesity has been identified, the fat mass and obesity associated gene, *FTO *[9-14]. The first association between *FTO *and human obesity was found by Frayling et al. in early 2007 [10] in a genome wide association study of diabetes and has been positively replicated in additional studies involving several different ethnicities [15-21] including Swedish subjects [22,23]. These studies all show that subjects who are homozygous for the risk allele weigh about 3 kg more compared to those without the allele and individuals with the risk allele have about 1.5-fold increased risk of having obesity.

Most of the previous studies on the *FTO *gene variants are cross-sectional, leaving the longitudinal pattern of the associations between obesity and age-specific genetic effects poorly studied. Furthermore, the genetic effects on lifestyle factors and the effect in populations not affected by an obesogenic environment have not been thoroughly investigated. There have also been additional studies indicating that the genetic effects on BMI may be dependent on physical activity [24-26]. Four studies have so far analyzed if the effect of *FTO *on obesity in adults may be changed upon physical activity [23,27-29] with somewhat contrasting results. The aim of the present study was to address the issues of BMI and physical activity by assessing the genetic effects in a longitudinal cohort of adult men, the ULSAM cohort. The subjects were all born between 1920 and 1924, investigated at the age of 50 and reinvestigated at the ages of 60, 70, 77 and 82 years of age. The data available in Uppsala Longitudinal Study of Adult Men (ULSAM) allowed us to, compared to previous studies, investigate BMI differences at older ages and also highlight that the environment may play a fundamental role in the genetic contribution to increased BMI.

## Methods

### Subjects

In 1970, all men born between 1920 and 1924 and residing in Uppsala, Sweden, were invited to participate in a health survey, the Uppsala Longitudinal Study of Adult Men (ULSAM), described previously [30]. At baseline, at age 50 years, 2841 men were invited and 82% (n = 2322) accepted to participate. The subjects were then re-invited for examination at the ages of 60, 70, 77, and 82 years. At age 60, 2130 men were invited and 87% (1860) participated. At age 70, 1681 men were invited and 73% (1221) participated. The fourth examination was performed when the men were aged 77 years. At this time 748 of the 2322 participants at age 50 had died and another 176 men were ineligible for other reasons, leaving 1398 men possible subjects and 60% (839) attended the examination. At the last examination, at 82 years of age 971 men were invited and of the invited, 530 men (56%) participated in the examination. This study was approved by the Ethics committee of Uppsala University, Faculty of Medicine. All participants gave their written informed consent.

### Anthropometry

The men were invited by a letter, which also explained the aim of the examination and all investigations were carried out under standardized conditions. Height was measured to the nearest whole centimeter and weight to the nearest whole kilogram. BMI was calculated as weight divided by height squared (kg/m2) and waist circumference in centimeter was measured midway between the lowest rib and the iliac crest in a supine position. The body fat was estimated by the measurement of skinfold thickness with a Harpenden caliper. The skinfold was measured to the nearest 0.2 mm on three different sides: on the back of the middle of the over-arm, just below the angle of the scapula and on the abdomen to the right of the umbilicus. All measurements were made with the subject in the sitting position.

### Physical activity

DNA and information on self reported leisure time physical activity at age 50 years was available on 1860 men. This was estimated using four questions in a medical questionnaire. Based on these questions four different physical activity categories was constructed: sedentary, moderate, regular and athletic as described and validated previously [31-33].

### Genotyping

Of the initial cohort of 2322 men, DNA was available from 1152 menobtained from examination at 70 and 77 years of age. The genotyping of *FTO *rs9939609 was carried out at the SNP technology platform at Uppsala University http://www.genotyping.SE/ using a Illumina Golden Gate Assay [34]. The SNP genotype call rate in the samples was 96.8%.

### Statistical analysis

In order to test for deviation from Hardy-Weinberg equilibrium the Person's χ^2 ^-test (1 d.f) was applied. Genotype and allele frequencies were calculated and logistic regression was used to calculate odds ratio (OR) with a 95% confidence interval (CI) assuming an additive model. Association with overweight and obesity was determined comparing subjects with normal weight (BMI < 25 kg/m^2 ^) and overweight (BMI ≥ 25 kg/m^2 ^) and subjects with normal weight (BMI < 25 kg/m^2 ^) and obesity (BMI ≥ 30 kg/m^2 ^), respectively. Quantitative skewed variables were log-transformed before analysis. Associations between genotypes and phenotypes were analyzed with linear regression, assuming an additive and dominant model. The effect of physical activity on the impact of the *FTO *rs9939609 on BMI levels was analyzed with an ANOVA test, assuming an additive model and an interaction parameter was included to test for interaction effects. Association between *FTO *rs9939609 and BMI across different age groups was analyzed with linear models and only subjects with available data at age 82 were included in the analysis. P-values < 0.05 were considered significant. All the analysis was performed using PLINK http://pngu.mgh.harvard.edu/purcell/plink/[35] and Graphpad Prism version 4.03 (PraphPad Software, San Diego, USA).

## Results

The obesity associated *FTO *variant rs9939609 was genotyped in the ULSAM cohort, a longitudinal cohort of adult men. Descriptive characteristics at baseline of the cohort are presented in Table 1. Of the total number of subjects 631 (55%) were considered normal weight, 521 (41%) had a BMI over 25 kg/m^2 ^and 55 (4.8%) had a BMI over 30 kg/m^2 ^and were considered overweight and obese, respectively. The mean BMI among all subjects was 24.8 ± 2.9 kg/m^2 ^and among the normal weight 22.8 ± 1.5 kg/m^2 ^. Among the overweight and obese the mean BMI was 27.3 ± 2.1 kg/m^2 ^and 32.1 ± 1.9 kg/m^2 ^, respectively.

**Table 1:** Descriptive characteristics of all subjects included in the analysis at 50 years.

Characteristics	All	Normal weight	Overweight	Obese
N	1152	631	521	55
Weight (kg)	77.4 ± 10.0	71.4 ± 6.8	84.7 ± 8.2	100.1 ± 10.3
Length (m)	176.5 ± 5 8	176.9 ± 5.8	176.0 ± 5.7	175.6 ± 6.4
BMI (kg/m^2 ^)	24.8 ± 2.9	22.8 ± 1.5	27.3 ± 2.1	32.1 ± 1.9

Values are geometric means ± SD.

The analysis on *FTO *rs9939609 showed an allele frequency similar as reported by Frayling et al [9] with a minor allele frequency of 37% among the normal-weight subjects, 40% and 37% among overweight and obese subjects, respectively. The allelic odds ratio for rs9939609 on the risk of being overweight and obese compared to being of normal weight was estimated comparing subjects with normal weight (BMI < 25 kg/m^2 ^) and overweight (BMI ≥ 25 kg/m^2 ^) and subjects with normal weight (BMI < 25 kg/m^2 ^) and obesity (BMI ≥ 30 kg/m^2 ^), assuming an additive model but no association was found in either groups (Table 2).

**Table 2:** Association study of *FTO *rs9939609 variant with overweight and obesity

Group	n	Genotype, n (%)			MAF, %	OR (95% CI)	P	HWE
						
		TT	TA	AA				
Normal weight	607	242 (40)	277 (46)	88 (14)	37			0.544
Overweight	454	169 (37)	206 (45)	79 (17)	40	1.124 (0.942-1.341)	0.194	0.242
Obese	54	16 (30)	29 (54)	9 (17)	44	1.294 (0.870-1.927)	0.203	0.588

Data are number of subjects in each group, at age 50 years, and number of subjects for each genotype (% in each group). Minor allele frequency (MAF) for each group is given in percentage. Odds ratio (OR) with a 95% confidence interval (CI) was calculated assuming an additive model. Association with overweight and obesity was determined comparing subjects with normal weight (BMI < 25 kg/m^2 ^) and overweight (BMI = 25 kg/m^2 ^) and subjects with normal weight (BMI < 25 kg/m^2 ^) and obesity (BMI ≥ 30 kg/m^2 ^), respectively. HWE indicate p-values for deviation from Hardy-Weinberg Equilibrium.

**Figure 1:**
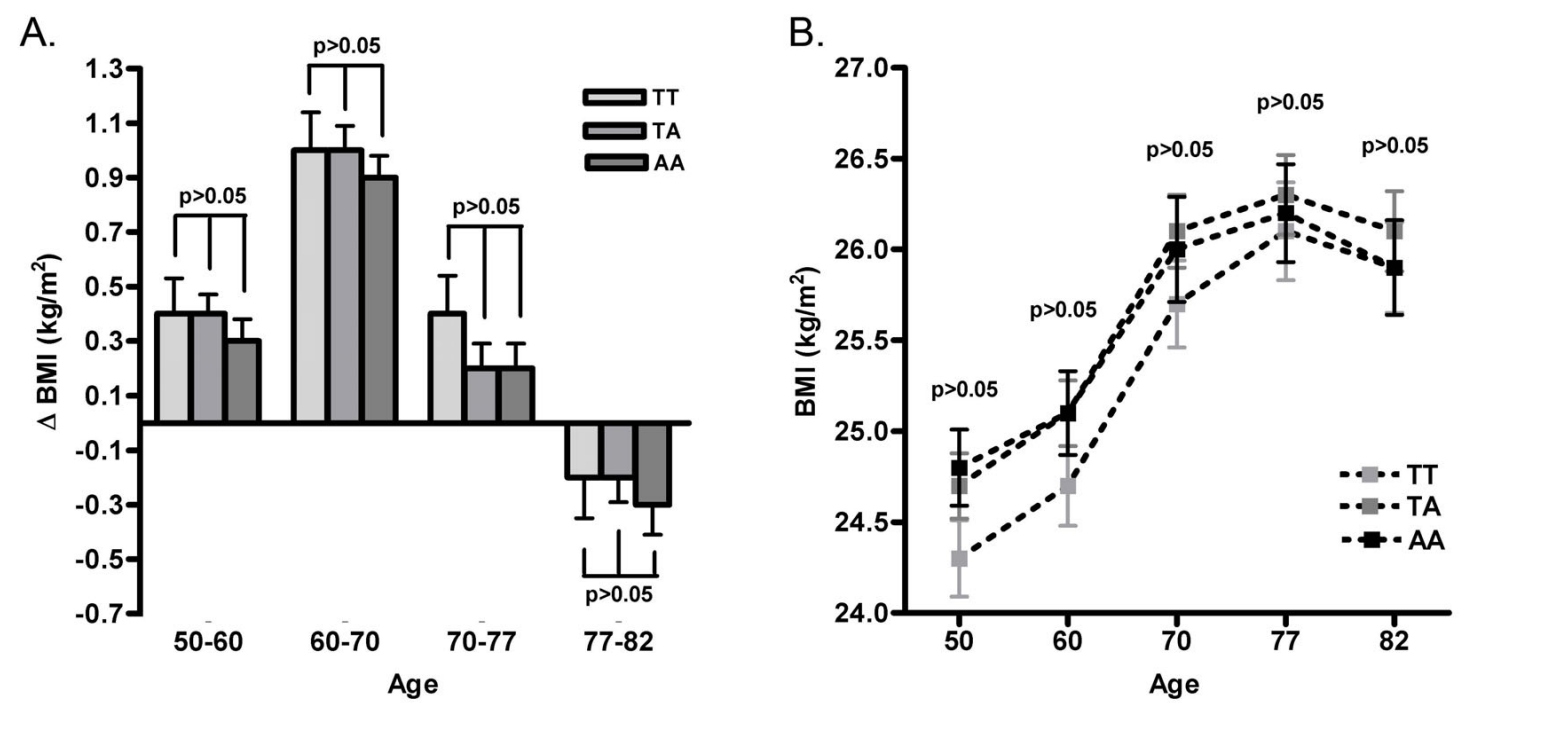
**A: Associations between *FTO *rs9939609 and BMI across different ages; 50, 60, 70, 77 and 82 years**. **B**: Changes in BMI levels between different ages (50-60, 60-70, 70-77 and 77-82 years) and according to *FTO *rs9939609 genotype.

We further investigated the association between rs9939609 and BMI as well as other measurements of obesity such as waist circumference and skinfold thickness at baseline for all subjects as well as BMI and waist circumference at 60, 70, 77 and 82 years of age. No association at any age was found between the minor A-allele and BMI, or any of the other obesity measurements (Table 3, Figure 1).

**Table 3:** Anthropometric characteristics stratified according to *FTO *rs9939609genotypes at different ages.

	TT	TA	AA	**P**_Add _	**P**_Dom _
**Age 50**					
N = 1115	426	512	177		
Weight (kg)	76.8 ± 9.4	77.8 ± 10.5	78.0 ± 10.7	0.309	0.192
Length (m)	176.1 ± 5.9	176.8 ± 5.5	176.5 ± 5.8	0.235	0.072
BMI (kg/m^2 ^)	24.3 ± 2.8	24.7 ± 2.9	24.8 ± 3.2	0.130	0.159
Waist circumference (cm)	86.0 ± 8.3	87.3 ± 8.6	88.6 ± 8.7	0.189	0.323
Skinfold abdominal (mm)	19.2 ± 9.2	19.5 ± 9.2	19.4 ± 9.5	0.538	0.949
Skinfold subscapular (mm)	16.2 ± 5.7	15.8 ± 5.6	17.0 ± 6.9	0.857	0.246
Skinfold triceps (mm)	10.2 ± 3.9	10.3 ± 3.8	10.2 ± 3.9	0.282	0.243

**Age 60**					
N = 1051	404	479	168		
Weight (kg)	77.4 ± 10.2	79 0 ± 11.0	78.9 ± 11.8	0.169	0.171
Length (m)	175.7 ± 5.9	176.4 ± 5.5	176.0 ± 5.9	0.303	0.121
BMI (kg/m^2 ^)	24.7 ± 2.9	25.1 ± 3.2	25.1 ± 3.4	0.147	0.111
Waist circumference (cm)	89.5 ± 9.5	90.3 ± 9.8	89.9 ± 10.2	0.383	0.159
Skinfold abdominal (mm)	26.7 ± 11.2	26.8 ± 10.5	26.7 ± 11.2	0.624	0.697
Skinfold subscapular (mm)	18.5 ± 7.1	17.7 ± 7.2	18.7 ± 7.1	0.739	0.586
Skinfold triceps (mm)	10.1 ± 5.2	10.4 ± 6.4	9.9 ± 5.7	0.879	0.978

**Age 70**					
N = 1056	401	490	165		
Weight (kg)	78.8 ± 10.2	81.1 ± 11.9	80.2 ± 12.7	0.842	0.814
Length (m)	174.6 ± 5.9	175.2 ± 5.6	174.9 ± 6.1	0.259	0.116
BMI (kg/m^2 ^)	25.7 ± 3.1	26.1 ± 3.5	26.0 ± 3.6	0.426	0.787
Waist circumference (cm)	94.0 ± 8.9	95.3 ± 9.8	94.7 ± 10.2	0.266	0.203

**Age 77**					
N = 749	287	342	120		
Weight (kg)	77.8 ± 11.2	79.8 ± 11.5	79.6 ± 11.9	0.813	0.804
Length (m)	173.5 ± 5.7	173.8 ± 5.6	173.5 ± 6.0	0.849	0.623
BMI (kg/m^2 ^)	26.1 ± 3.5	26.3 ± 3.5	26.2 ± 3.6	0.146	0.085
Waist circumference (cm)	94.9 ± 9.7	95.9 ± .9.9	96.1 ± 10.7	0.366	0.341

**Age 82**					
N = 493	193	230	70		
Weight (kg)	77.4 ± 10.8	78.2 ± 11.3	76.0 ± 11.7	0.548	0.963
Length (m)	173.3 ± 5.6	173.0 ± 5.6	171.9 ± 5.1	0.102	0.295
BMI (kg/m^2 ^)	25.9 ± 3.5	26.1 ± 3.3	25.9 ± 3.7	0.259	0.225
Waist circumference (cm)	95.8 ± .9.7	95.6 ± 9.3	95.7 ± 11.2	0.902	0.851

Values are geometric means ± SD. Skewed variables were log-transformed and analyzed with linear regression, assuming an additive and dominant model.

The longitudinal pattern of the associations between obesity and age-specific genetic effects was analyzed in 32-years follow up. Although not significant, we observed a trend toward decreasing differences in BMI according to genotype at older ages, as was also observed by [36](Figure 1A). We also investigated the longitudinal changes in BMI from the age of 50 years to 82 years (Figure 1B). We observed an increase in BMI levels from 50-77 years, with the highest increase between 60-70 years, and a decrease in BMI from 77-82. However, neither the A or T allele at any age were associated with a higher increase or decrease in BMI which means that the variant does not seem to affect the changes in BMI levels at any stage in older life.

**Figure 2:**
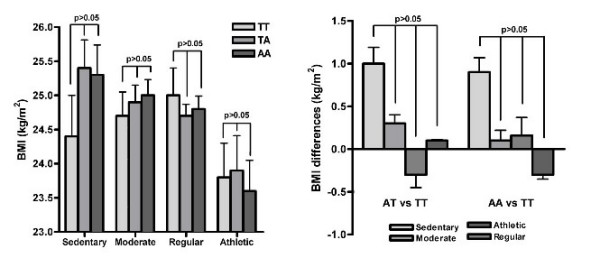
**Effect of physical activity on the impact of the *FTO *rs9939609 variant on BMI levels at age 50 years**. Subjects were divided into four different categories based on self-reported leisure time physical activity: sedentary, moderate, regular and athletic. **A**: BMI levels for each category stratified according to *FTO *rs9939609 genotype, data are means and ± SEM **B**: Differences in BMI levels for the different categories between heterozygotes and homozygotes T-allele carriers and between heterozygotes and homozygotes A-allele carriers, respectively.

From a medical questionnaire we had access to self-reported leisure time physical activity at age 50 years which divided the subjects into four categories. 15% of all subjects were considered sedentary, 36% had moderate physical activity, 44% had regular physical activity and 5% were considered athletic, and as expected subjects that were considered athletic had lower BMI levels compared to the less active subjects (p < 0.01). The subjects, based on the physical activity, were further stratified according to the *FTO *rs9939609 genotype (Figure 2A). Physical activity influence the effect of *FTO *rs9939609 on BMI (P_interaction _= 0.039) across the four different categories. The BMI per FTO risk allele was higher in sedentary active (0.33 per risk allele, SD 0.39) and moderate active individuals (0.13 per risk allele, SD 0.21) compared with the BMI among individuals with regular activity (-0.25 per risk allele, SD 0.18) and athletic individuals (-0.29 per risk allele, SD 0.47). The difference in BMI per allele in each category was however not significant different (P > 0.05 for all categories) (Figure 2B).

## Discussion

There has been a considerable increase in obesity from 6% in 1963 to 11% in 1993 in Sweden [2,37]. This increase has continued and there has been a twofold increase during the last two decades [3,4,38]. The men included in the ULSAM cohort were all examined 1970-74 at the age of 50 years, when the prevalence of moderate obesity were very low, 0.8% compared to 3.8% in 2000-2005 [4]. Moreover, these men have lower BMI levels compared to the cohorts previously used to study the FTO gene. The mean BMI was 24.5 in the ULSAM cohort compared to the mean BMI of 26.5 in the cohorts of older adult reported by Frayling et al. [9]. The ULSAM cohort provides thus an important opportunity to study genetic components in a lean population that lived in a less obesogenic environment compared with today. Our results indicate that the impact of *FTO *seems less prominent in this lean population since there was no significant difference in BMI for the carriers of the risk allele, not at baseline or at any of the other ages that we studied. Neither did we find any association for other measurements of obesity such as waist circumference and skinfold thickness at any age. It is however important that these findings are replicated in several lean populations in order to be able to draw any conclusions.

We further analyzed the longitudinal pattern of the associations between BMI and *FTO *variants. So far, Frayling et al. [9] and Qi et al. [36] have observed a trend toward decreasing associations between *FTO *variants and BMI at older age. Qi et al. further showed that this was found in men, whereas the associations were constant across different age groups in women [36]. Although we did not reach significance, our data support these findings. The small effect of rs9939609 on BMI in our cohort at baseline was not seen at all at older ages. Thus, it appears that the association between the SNP and obesity risk declined with older age especially in men.

Studies on BMI changes in relation to physical activity and genetic factors have shown that both play a significant independent role in weight changes. However, there has been additional evidence of a gene-environment interaction in men, indicating that genetic effects on weight gain may be more highly dependent on physical activity level [24-26]. Four studies have so far analyzed if the effect of *FTO *on obesity in adults may be changed upon physical activity [27,23,29] with somewhat contrasting results. Andreasen et al. [27] demonstrated that the impact of the *FTO *rs9939609 genotype was influenced by the habitual level of physical activity in a population based study on Danish subjects with mean BMI of 26 kg/m^2 ^. These subjects were divided according to self-reported physically activity into passive, light or medium physically active and hard or very hard physically active, and stratified according to *FTO *genotype. The study showed that physical inactive homozygous *FTO *rs9939609 A-allele carriers had around 2 kg/m^2 ^higher BMI compared to inactive homozygous T-allele carriers [27]. These findings were supported by Cauchi et al [29] and Rampersaud et al. [28] for other *FTO *variants. Both showed that the differences in BMI were larger in physical inactive subjects while no difference was found at all in physical active subjects. However, a large study by Jonsson et al. [23] on Swedish adults did not find any interaction between rs9939609 and physical activity on BMI. The biggest differences between these studies is that the Swedish subjects had a mean BMI of 24.3 kg/m^2 ^and were leaner compared to the other cohorts and physical activity was only based on two categories, active or not active. Our results showed the presence of an interaction and support the suggestion that the increased levels of BMI related to *FTO *variants may be less prominent if the persons are physical active. Our interaction is however weaker compared to the other studies and the effect size is smaller. The lower effect size in our study compared to previous studies may be due to a leaner cohort. We have furthermore older subjects compared to other studies which may affect the interaction and weaken the effect of physical activity. Furthermore, since the categories of physical activity in our study have been assessed by questionnaire under- or over-reporting may occur which may also influence the result. Our finding is however consistent with previous studies and suggests that inherited factors significantly influence body weight in sedentary subjects and that in physically more active individuals the effect of the FTO gene seem to diminished, further suggesting that high physical activity would be particularly beneficial in those genetically predisposed to obesity due to the *FTO *gene. As the gene expression might vary due to differences in the environment it is interesting to study the effect of *FTO *in a population with lower mean BMI than has previously been studied. Furthermore, several of the susceptibility genes to common diseases do not have a primary causal role in the predisposition to disease without considering environmental factors. Rather, susceptibility genes may act as responders, or modifiers, to factors such as stress, environment, as well as physical inactivity. Fisher et al. [39] has also demonstrated that loss of the mouse *Fto *gene product reduces adiposity, and that even moderate reduction in *Fto *expression protects from diet-induced obesity which makes it tempting to speculate that physical activity may reduce the *FTO *gene expression and influence the interacting genes that in turn modify energy homeostasis.

## Conclusion

Here we set out to examine the association of the *FTO *variant rs9939609 with BMI in a longitudinal cohort of adult men. We could not find any association between *FTO *variant and obesity or obesity measurements at any of the ages studied. We further analyzed the effect of physical activity on BMI levels and found an interaction between rs9939609 and level of activity and saw that inactive people carriers of the A-allele had higher BMI-values than non-carriers. This, together with our lack of association with BMI in our lean population, indicate that *FTO *seem to have a greater effect on those that are less physical active and with higher BMI. Thus, these results are an example of environmental influences and that inactivity is a potential vital environmental trigger for disease susceptibility dependent on the *FTO *genotype.

## Competing interests

The authors declare that they have no competing interests.

## Authors' contributions

JJ carried out the study, analyzed the data, participated in the design, and drafted the manuscript. TA performed the genotyping, participated in the genetic analysis and in the writing of the manuscript. UR and LL participated in analysis of the phenotypes and in writing of the manuscript. RF and HS conceived the study, participated in the design and writing of the manuscript. All authors read and approved the final manuscript.

## Pre-publication history

The pre-publication history for this paper can be accessed here:

http://www.biomedcentral.com/1471-2350/10/131/prepub
